# The Noncell Autonomous Requirement of Proboscipedia for Growth and Differentiation of the Distal Maxillary Palp during Metamorphosis of* Drosophila melanogaster*

**DOI:** 10.1155/2017/2624170

**Published:** 2017-03-05

**Authors:** Anthony Percival-Smith, Gabriel Ponce, Jacob J. H. Pelling

**Affiliations:** Department of Biology, University of Western Ontario, London, ON, Canada N6A 5B7

## Abstract

The* Drosophila* maxillary palpus that develops during metamorphosis is composed of two elements: the proximal maxillary socket and distal maxillary palp. The HOX protein, Proboscipedia (PB), was required for development of the proximal maxillary socket and distal maxillary palp. For growth and differentiation of the distal maxillary palp, PB was required in the cells of, or close to, the maxillary socket, as well as the cells of the distal maxillary palp. Therefore, PB is required in cells outside the distal maxillary palp for the expression, by some mechanism, of a growth factor or factors that promote the growth of the distal maxillary palp. Both* wingless (wg)* and* hedgehog (hh)* genes were expressed in cells outside the distal maxillary palp in the lancinia and maxillary socket, respectively. Both* wg* and* hh* were required for distal maxillary palp growth, and* hh* was required noncell autonomously for distal maxillary palp growth. However, expression of* wg-GAL4* and* hh-GAL4* during maxillary palp differentiation did not require PB, ruling out a direct role for PB in the regulation of transcription of these growth factors.

## 1. Introduction

The life cycle of* Drosophila* has two distinct free-living forms: the larva and adult. During embryogenesis a larva is formed, and during the larval stages and metamorphosis the imaginal cells proliferate and differentiate to form an adult. The head of the larva and adult fly are highly derived relative to the archetypical insect head [1]. The important function of the mouthparts in adapting to distinct ecological niches [2] explains the large diversity of morphology of mouthparts in insects. The morphogenesis of the adult* Drosophila* mouthparts, the maxillary palpus and proboscis, requires four* Hox* genes:* labial (lab)*,* Deformed (Dfd)*,* pb*, and* Sex combs reduced (Scr)* [3–7]. The diversity of the structure and function of insect mouthparts observed during evolution of the lineages leading to* Drosophila*,* Tribolium*, and* Oncopeltus* is reflected in distinct requirements of HOX proteins for mouthpart development. The requirements of LAB, PB, DFD, and SCR in maxillary palpus development and the maxillary palpus phenotype due to the loss of these HOX proteins are distinct in* Drosophila*,* Tribolium*, and* Oncopeltus* [5, 6, 8, 9]. Even within the* Drosophila* life cycle, the requirements of HOX proteins for mouthpart development are distinct [10]. During embryogenesis PB is expressed in, but not required for, mouthpart development; SCR patterns the labial segment and DFD patterns the maxillary segment [11]. In adults, PB is required for patterning the maxillary palpus and PB with SCR is required for patterning the proboscis [12, 13].

The* Drosophila* maxillary palpus is a highly derived sensory appendage. The establishment of the adult maxillary palpus developmental field requires temporal regulation of wingless (WG) expression during the larval stages [14]. Although DFD expression during second and third stadium larvae defines a maxillary field, it is the delayed expression of WG that specifies maxillary palpus versus antennal identity. Precocious expression of WG in the maxillary primordia results in a maxillary palpus to antenna homeotic transformation. The maxillary palpus has a proximal-distal axis. Proximal-distal axis formation of the legs is well described in* Drosophila* [15, 16]. In the first step, the anterior and posterior compartments are established by the expression of Engrailed (EN) and Hedgehog (HH) in the posterior compartment. HH activates the expression of Decapentaplegic (DPP) in a sector of dorsal cells and the expression of wingless (WG) in a sector of ventral cells. The expression of the DPP and WG morphogens patterns the proximal-distal axis by regulating the expression of genes such as* Distalless (Dll)* and* homothorax (hth)* [16].

Determination and differentiation is easy to observe during embryogenesis and larval imaginal disc development but not during metamorphosis, because the pupae are opaque, the larval tissue is undergoing histolysis and the developing imaginal tissue is fragile. Although easy to identify body parts that have undergone overt differentiation in fixed pupal material, undifferentiated cells are hard to assign an origin and future. Finally dynamic temporal changes in gene expression are hard to identify by comparing one static, fixed and dissected pupal stage against another. The development of live imaging of metamorphosis allows access to the events of metamorphosis [17, 18]. In this paper, we show that PB is required noncell autonomously for growth of the distal maxillary palp but not by regulation of the transcription of two growth factor genes* wingless (wg)* and* hedgehog (hh)*.

## 2. Materials and Methods

### 2.1. *Drosophila* Stocks and Crosses

The fly strains were maintained on standard medium. All genotypes were generated by standard* Drosophila* crosses. The stocks used in this study are listed in Table 1.

### 2.2. Immunolocalization of PB/SCR and Detection of GFP/RFP

Staged prepupae and pupae were dissected from the pupal case in* Drosophila* Ringer's solution, and the pupal membrane was torn along the dorsal side of the thorax to allow penetration of the fixative. The prepupae and pupae were fixed for 20 min in PBS and 4% formaldehyde. Pupae were dissected further to remove more of the pupal membrane and histolysed larval tissue, and refixed for 20 min. For immunolocalization, rabbit anti-PB E9 polyclonal antibody and mouse anti-SCR monoclonal antibody were used to detect PB and SCR expression [24, 25]. The primary antibodies were visualized with donkey FITC conjugated anti-rabbit and Texas-red conjugated anti-mouse antibodies (Jackson laboratories). In the case of detection of GFP and RFP, fixed material was counterstained with DAPI. Images were collected on a Zeiss confocal microscope in the Biotron integrated microscopy facility. Detection of *β*-galactosidase activity was performed with the X-gal substrate using conditions as described in [26].

### 2.3. Live Imaging of Metamorphosis

Similar methods were used as described in [17] with one important modification: white prepupae were suspended in a small drop of halocarbon oil on a coverslip of a humidity chamber to improve greatly the live imaging of metamorphosis. Images of the time lapse were collected at approximately 2.53 minute intervals with a Hamamatsu digital camera mounted on a Leica DMRBE microscope and 2.5 minute intervals with a Zeiss confocal microscope. All images were exported as.tiff files, and to preserve relative intensities, all images of the time lapse were simultaneously adjusted for brightness, contrast and size with Adobe Photoshop. For the experiments with* hh-GAL4* and* wg-GAL4* in wild type and* pb*^*27*^*/pb*^*20*^ pupae, the pupae were mounted side by side during image capture, and after adjustment for intensity, the image of each pupa of the set was separated and used to make a movie in Adobe After Effects. These movies were synchronized in Adobe After Effects such that head eversion of the pupa occurred in the same frame. A movie of the appropriate time stamp was imported into Adobe After Effects and also synchronized to head eversion. The final movie was rendered and compressed in Adobe After Effects and exported as a QuickTime (MPEG4) file.

### 2.4. Phenotypic Analysis of* Drosophila* Heads

Heads were dissected from eclosed or pharate adults. For bright field microscopy the heads were incubated with 80% acetic acid 20% glycerol overnight at 60°C. The heads were mounted on slides in 1 : 1 Hoyer's mountant : lactic acid [27]. For scanning electron microscopy, the heads were critical point dried and sputter gold coated. Images were collected on a Hitachi 3400-N variable pressure scanning electron microscope in the Biotron integrated microscopy facility.

### 2.5. Mosaic Analyses

Flip-mediated mitotic recombination was used to generate all clones of mutant tissue [28]. Larvae were heat shocked for 1 h at 36.5°C. For marking the clones on the adult cuticle, either the* FRT82B Sb M y*^*+*^,* FRT82B pb*^*20*^* Sb M y*^*+*^ or* FRT82B M y*^*+*^*exd*^*+*^ chromosome were used screening for Sb^+^ M^+^ y^+^ cells (Table 4) [12, 19]. The* pb*^*27*^ clones in pupae expressing RFP driven by the* hh-GAL4* driver were generated in the genotype* y w; P*{*UASRFP*}*/P*{*hspFLP*}*; P*{*UAStrc*^*S292A T453A*^}*, FRT82B pb*^*27*^*hh-GAL4/FRT82B P*{*UbiGFP*} and homozygous* pb*^*27*^ cells identified by lack of expression of GFP.

### 2.6. Marking Adult Cuticle for Expression of* hh-GAL4*

The reporter* P*{*UAStrc*^*S292A T453A*^} was used [29]. Cells that express TRC^S292A  T453A^ had multiple stunted tricombs that were detected with a scanning electron microscope.

### 2.7. RNAi Reduction of Expression

RNAi lines were obtained from Vienna Drosophila RNAi Center and virgin females crossed with* w*^*1118*^*, P*{*UASdicer2, w*^*+*^}*; P*{*pb-GAL4, w*^*+*^}*, P*{*UASlacZ, w*^*+*^}*/CyO* males (VDRC60010 X CB10) [20, 21]. The crosses were reared at 29°C. The heads were mounted in Hoyer's mountant [27], and the length of the maxillary palps measured in Openlab 3.1. Five independent biological replicates were set up, and the mean lengths of male and female distal maxillary palps from the replicates were analyzed with an ANOVA for statistical significance in SSPS v. 16.0.

## 3. Results

### 3.1. The Requirement of PB for Maxillary Palpus Development

**Figure 1:**
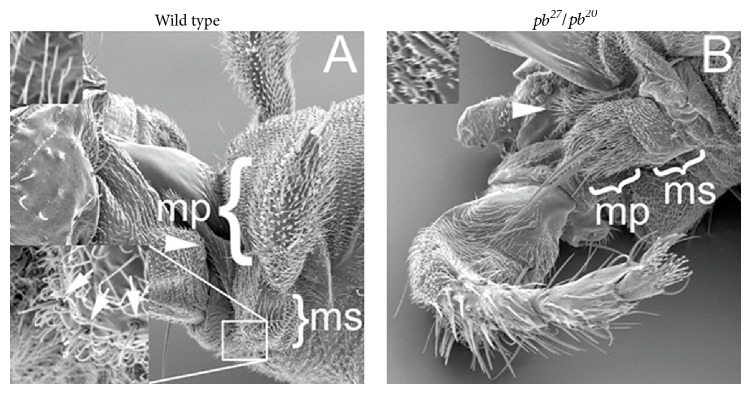
The proboscipedia phenotype. In both panels the ventral side is on the left. Panel (A) is wild type mouthparts. The mp bracket indicates the distal maxillary palp and the ms bracket indicates the proximal maxillary socket. The insert at the bottom left is a close-up of the portion of the maxillary socket with three proximal palpus bristles indicated by the arrows. Panel (B) is a* pb*^*27*^*/pb*^*20*^ transformed mouthpart with the reduced distal maxillary palp (mp) and reduced proximal maxillary socket (ms) indicated with brackets. The arrowheads indicate the lancinia in both panels. The inserts on the top left of each panel show the long tricombs found on the maxillary socket of wild type (panel A) and the short tricombs found on the maxillary socket of* pb* mutants (panel B).

**Figure 2:**
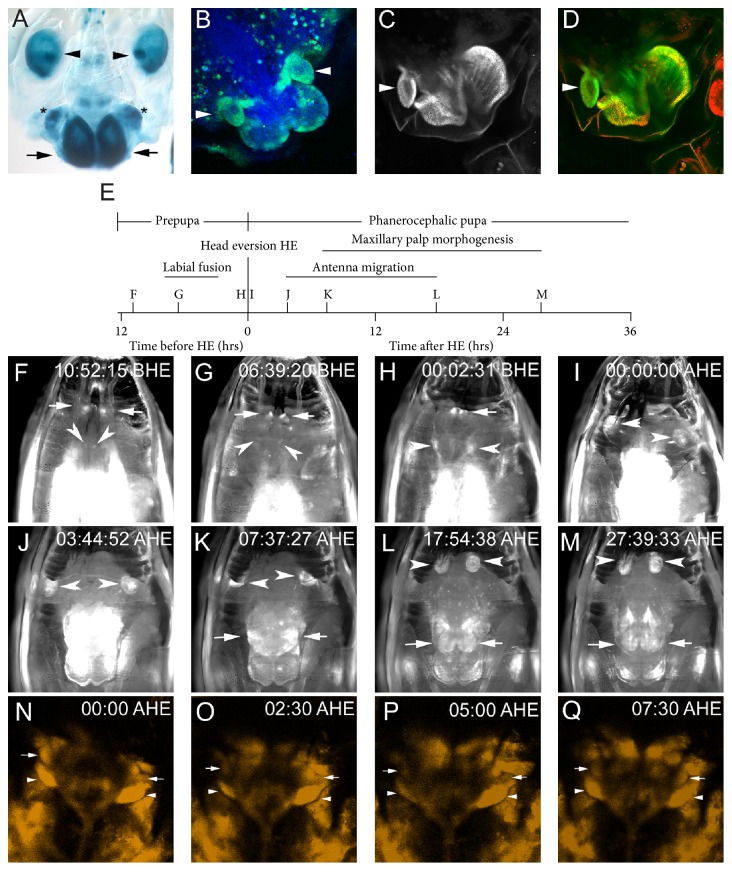
The expression of PB and* pb-GAL4* during metamorphosis. Panel (A) is the expression of* pb-GAL4* recorded using the* UASlacZ* reporter gene fixed just before head eversion. The arrows indicate the fused labial discs, asterisks indicate the maxillary palp primordia, and the arrowheads show the antennal primordia. Panel (B) is the expression of* pb-GAL4* in developing mouthparts (approximately 18 h AHE) using* UASEGFP* as the reporter gene (green). The arrowheads indicate the distal maxillary palps. The tissue is stained with DAPI (blue). Panels (C) and (D) are the expression of PB (C) and the expression of PB (green) and SCR (red) (D) at approximately 36 h AHE. The arrowheads indicate the distal maxillary palp. Panel (E) is the time line of metamorphosis indicating the stages and major events observed. The start and stop point for labial fusion, antenna migration, and maxillary palp morphogenesis are estimates based on first evidence of movement. The letters indicate the relative time of the images shown in panels (F)–(M). Panels (F)–(M) are individual frames from live imaging shown in Supplemental Data Movie 1. The time the image was recorded is indicated (h: min: sec BHE or AHE) and the arrows indicate the developing labial segment and the arrowheads indicate the aristal primordia and antennal primordia expression. Panels (N)–(Q) are the first 7.5 minutes AHE of YFP expression driven by* pb-GAL4* shown in Supplemental Data Movie 2. The arrowheads indicate the proboscis primordia and the arrows the maxillary palpus primordia. In panels (A)–(D) and (I)–(Q) the dorsal side of the head is at the top and the ventral is at the bottom.

The maxillary palpus and the proboscis constitute the mouthparts of* Drosophila*. The maxillary palpus is composed of two pieces: the distal maxillary palp, the mobile sensory appendage, and the proximal maxillary socket into which the distal maxillary palp is inserted (Figure 1(A)). The formation of the adult mouthparts required the* Hox* gene* pb* (Figure 1), and PB protein was specifically expressed in the developing maxillary palpus and proboscis (Figures 2(C) and 2(D)). The HOX protein SCR, required for proboscis development but not required for maxillary palpus development, was expressed in the developing proboscis (Figure 2(D)). PB was expressed in both the cells of the distal maxillary palp and the cells surrounding the distal maxillary palp. This latter expression of PB outside the distal maxillary palp initiated a close examination of the* pb* null phenotype.

The cells surrounding and outside the distal maxillary palp primordium give rise to the proximal maxillary socket and lancinia. In null* pb*^*27*^*/pb*^*20*^ mutant adults both the distal maxillary palp and the proximal maxillary socket were reduced, but the lancinia was unaffected (Figure 1(B)). The reduction of the proximal maxillary socket was also associated with the loss of the proximal palpus bristles and the 7.5 fold reduction of tricomb length from 9.64 ± 0.77 *μ*m to 1.28 ± 0.09 *μ*m (±SEM, *n* = 12) on maxillary socket cells [30] (Figures 1(A) and 1(B)). Therefore, cells surrounding the distal maxillary palp were also affected by loss of PB expression.

### 3.2. Live Imaging of Maxillary Palpus Development

Live imaging of metamorphosis was employed to observe mouthpart development (Figure 2; Supplemental Data Movies 1 and 2 in Supplementary Material available online at https://doi.org/10.1155/2017/2624170) [17]. The prepupal and planerocephalic stages of metamorphosis were recorded, and head eversion is the boundary between the two stages. For all live imaging the first image after head eversion (AHE) is time zero of the pupal planerocephalic stage, and the time during the prepupal stage leading up to head eversion is before head eversion (BHE) (Figures 2(H) and 2(I)). To mark mouthpart development, a* pb-GAL4* driver was used to drive expression of GFP or YFP [20].* pb-GAL4* has many sites of ectopic expression in addition to expression in the mouthparts [20]. In wandering third stadium larvae,* pb-GAL4* is expressed in a small ring of cells in the aristal primordia, which was observed in the early prepupae (Figure 2(F)). In addition,* pb-GAL4* is expressed in the wings, legs, larval salivary glands, brain, and peripheral nervous system (Figures 2(F)–2(M)); Supplemental Data Movie 1).

Live imaging revealed that the levels and pattern of* pb-GAL4* expression were dynamic during metamorphosis. During the prepupal stage,* pb-GAL4* was expressed strongly in the salivary glands, brain, PNS (Keilin's organs and an anterior sensory complex potentially the labial sensory organ), and labial imaginal discs (Figure 2(F)). The major event observed with* pb-GAL4* during the prepupal stage important for mouthpart development was the fusion of the labial imaginal discs, which was associated with strong expression of* pb-GAL4* (Figures 2(A), 2(G), and 2(H)). Before head eversion, the imaginal tissue of the eye antennal, clypeolabral, and labial discs had fused, and* pb-GAL4* was expressed strongly in the fused labial discs, in the antenna, and in the primordia of the maxillary palpus (Figure 2(A)). However, we were unable to detect* pb-GAL4* expression in the maxillary palpus during the prepupal stage in live imaging, and therefore, do not know exactly when* pb-GAL4* is first expressed in the maxillary palpus.

After head eversion, differentiation of the mouthparts continues forming readily identifiable mouthparts (Figure 2(M)). Expression of* pb-GAL4* in the mouthpart primordia was obscured for a few hours after head eversion by the histolysis of the salivary glands in the movie using fluorescence optics (Figure 2(J)). Using confocal microscopy,* pb-GAL4* expression was detected in the maxillary palp and proboscis primordia after head eversion (Figures 2(N)–2(Q) Supplemental Movie 2). As the GFP/YFP signal expressed from the larval salivary glands degraded, two bright spots of* pb-GAL4* expression appeared in the mouthpart primordia (Figure 2(K), Supplemental Movies 1 and 2). The expression of* pb-GAL4* intensified in the two spots and the cells of the developing proboscis became more visible (Figure 2(l)). A bright spot of* pb-GAL4* expression appeared to be pushed dorsally during the differentiation of distal maxillary palps. Distal maxillary palp growth occurred between 7:37 and 27:40 h AHE. The bright spot of GFP expression was in cells of the maxillary socket (Figure 2(B)). In the mouthparts, the* pb-GAL4* driver reproduces the expression pattern of PB well (Figures 2(B), 2(C), and 2(M)). Importantly both* pb-GAL4* and PB are expressed in cells surrounding the distal maxillary palp as well as the cells of the developing distal maxillary palp.

Antennal differentiation was illuminated by ectopic expression of* pb-GAL4*. Just before head eversion the expression of* pb-GAL4* in the antenna went from a small circle in the arista primordia to throughout the antenna becoming more intense (Figure 2(H), Supplemental Movie 1). After head eversion the expression of* pb-GAL4* was very strong (Figure 2(I)). Between 3:45 and 17:55 h AHE, the antenna continued differentiation and migrated toward the centerline with the ongoing differentiation of the head.

### 3.3. Noncell Autonomous Requirement of PB for Growth of the Distal Maxillary Palp

**Figure 3:**
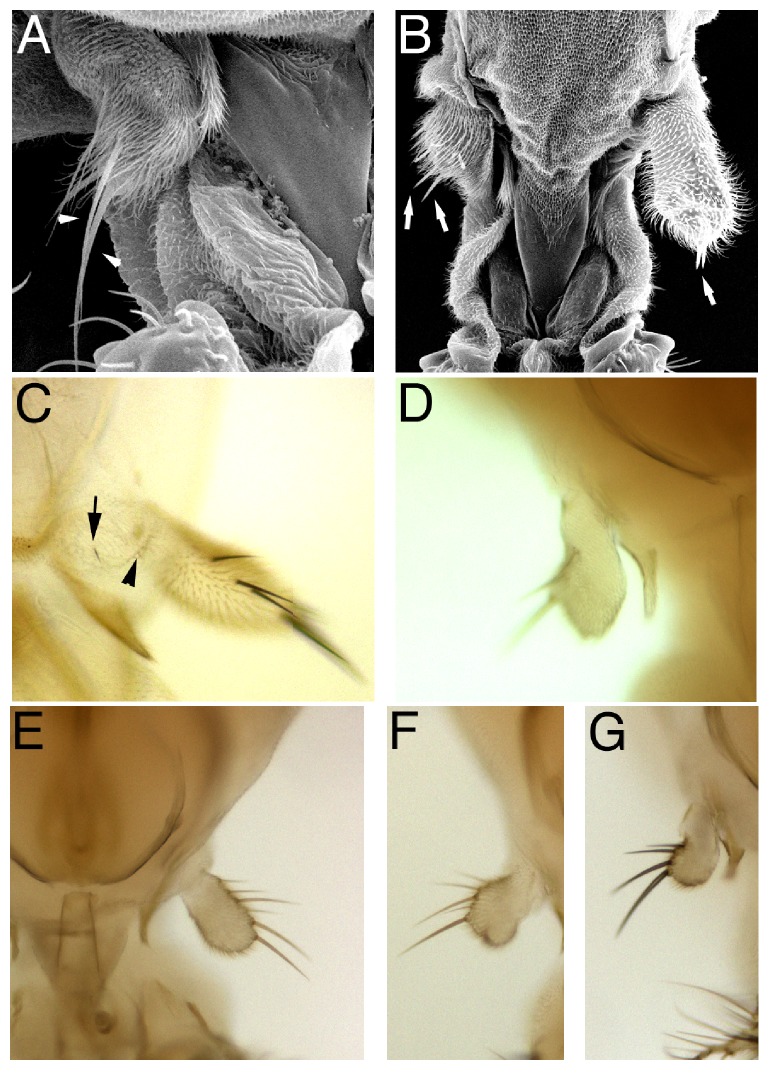
The maxillary palpus phenotypes of three independent mosaic analyses. Panels (A) and (B) are scanning electron micrographs of the effects of clonal loss of PB function generated in flies with the genotype* y w; P*{*hspFLP*}*/*+*; P*{*ry*^*+*^*, neo*^*r*^*, FRT*}*82B pb*^*27*^/*P*{*ry*^*+*^*, neo*^*r*^*, FRT*}*82B Sb*^*63b*^* M(3)95A*^*2*^* P*{*y*^*+*^*, ry*^*+*^}*96E*. In panel (A), a Sb^+^* pb*^*27*^ clone in the distal maxillary palp is shown and the maxillary palp is reduced. In panel (B), Sb* pb*^*+*^ distal maxillary palps are shown; the right is wild type, and the left is reduced indicated by the arrow. In panels (A) and (B), the arrows indicate Sb bristles and the arrowheads Sb^+^ bristles. Panels (C) and (D) are bright field micrographs of clonal loss of PB function generated in the genotype* y w; P*{*hspFLP*}*/*+*; P*{*ry*^*+*^*, neo*^*r*^*, FRT*}*82B pb*^*27*^* Scr*^*2*^* P*{*w*^*+*^*, ry*^*+*^}*90E*/*P*{*ry*^*+*^*, neo*^*r*^*, FRT*}*82B M(3)95A*^*2*^* P*{*y*^*+*^*, ry*^*+*^}*96E P*{*exd*^*+*^*, w*^*+*^}. Panel (C) is one of the 22* pb*^*27*^/+ wild type distal maxillary palps with the y^+^ proximal palpus bristle indicated by the arrow and y^+^ maxillary socket tricombs indicated with the arrowhead. Panel (D) shows a y^−^* pb*^*27*^*Scr*^*2*^ maxillary palpus. Panels (E)–(G) are bright field micrographs of distal maxillary palps from the clonal ectopic expression of PB in a* pb*^*27*^*/pb*^*20*^ mutant background generated in the genotype* y w, P*{*w*^*+*^*, pb*^*a*^*>y*^*+*^*>Tubα1*}*B; P*{*hspFLP*}*/*+*; pb*^*27*^*/pb*^*20*^. Panel (E) is a rescued y^−^ and PB expressing (*pb*^*a*^*>Tubα1*) maxillary palpus, panel (F) is a reduced y^−^ and PB expressing (*pb*^*a*^*>Tubα1*) maxillary palpus, and panel (G) is a reduced y^+^* pb*^−^ (*pb*^*a*^*>y*^*+*^*>Tubα1*) maxillary palpus.

Using FLP-mediated mitotic recombination to generate genetically mosaic flies with clones of* pb*^*27*^ mutant cells showed that PB is required noncell autonomously in cells of, or close to, the proximal maxillary socket for distal maxillary palp growth, as well as being required in the cells of the distal maxillary palp for growth [12, 28]. All* pb*^*27*^ clones (Sb^+^ M^+^) in the distal maxillary palp were reduced (Figure 3(A)) (Table 2), suggesting that PB is required in the distal maxillary palp for growth. Interestingly though, one-quarter of the Sb M* (pb*^*+*^) distal maxillary palps were also reduced, suggesting that PB is also required noncell autonomously in cells outside the distal maxillary palp for growth (Figure 3(B)) (Table 2). To determine which cells outside the distal maxillary palp PB was required in, a second mosaic analysis using* FRT pb*^*27*^* Scr*^*2*^ and* FRT M y*^*+*^* exd*^*+*^ chromosomes was performed scoring the y^+^ phenotype of the tricombs and maxillary socket bristles [19]. In vestigial maxillary palps the tricombs of the socket cells were reduced 7.5 fold in length and were too small to assess the y^+/-^ phenotype (Figures 1(A) and 1(B)). Of the 189 maxillary palps examined in genetically mosaic flies, 22 had y^+^ wild type distal maxillary palps (Figure 3(C)). In all 22 of these examples, the maxillary socket cells were* (pb*^*+*^), as both the tricombs and maxillary socket bristles had the y^+^ phenotype, suggesting that PB expressed in the ectoderm cells of, or very close to, the maxillary socket is required for growth of the distal maxillary palp.

A potential hypothesis for the noncell autonomous role of PB is that PB expressed in the proximal maxillary socket cells is required for transcription of the* pb* gene in the distal maxillary palp. A simple model for this hypothesis is that PB expression in the proximal maxillary socket cells is required for the expression of a secreted factor that binds and acts on the distal maxillary palp cells to induce transcription of the* pb* gene, and this expression of PB in the distal maxillary palp cells directs growth and differentiation. A mosaic analysis with PB expressed from a* Tubulin α1 pb* fusion gene was used to test this hypothesis [12]. Flip recombinase was used to excise the* y*^*+*^ gene from a* Tubα1>y*^*+*^*>pb*^*a*^ construct (>*FRT* site) to create a* Tubα1> pb* fusion gene expressing PB in a* pb*^*27*^*/pb*^*20*^ mutant background. In all y^+^ distal maxillary palps, which do not express PB from the* Tubα1 pb* fusion gene, the distal maxillary palp was reduced indicating that PB expression in the distal maxillary palp cells is required for rescue (Figure 3(G)). 87% of the y^−^ and PB expressing distal maxillary palps were rescued (Figure 3(E)). But 13% of the y^−^ and PB expressing distal maxillary palps were not rescued, suggesting that expression of PB in the distal maxillary palp is not sufficient to rescue growth. (Figure 3(F)) (Table 3). This was the same phenomena observed with the generation of* pb*^*27*^ clones, and more importantly if PB is required noncell autonomously for* pb* transcription in the distal maxillary palp, then all distal maxillary palps with PB being expressed from the* Tubulin α1* promoter would have been rescued.

### 3.4. Expression of Wingless, Decapentaplegic, and Hedgehog during Maxillary Palpus Differentiation

**Figure 4:**
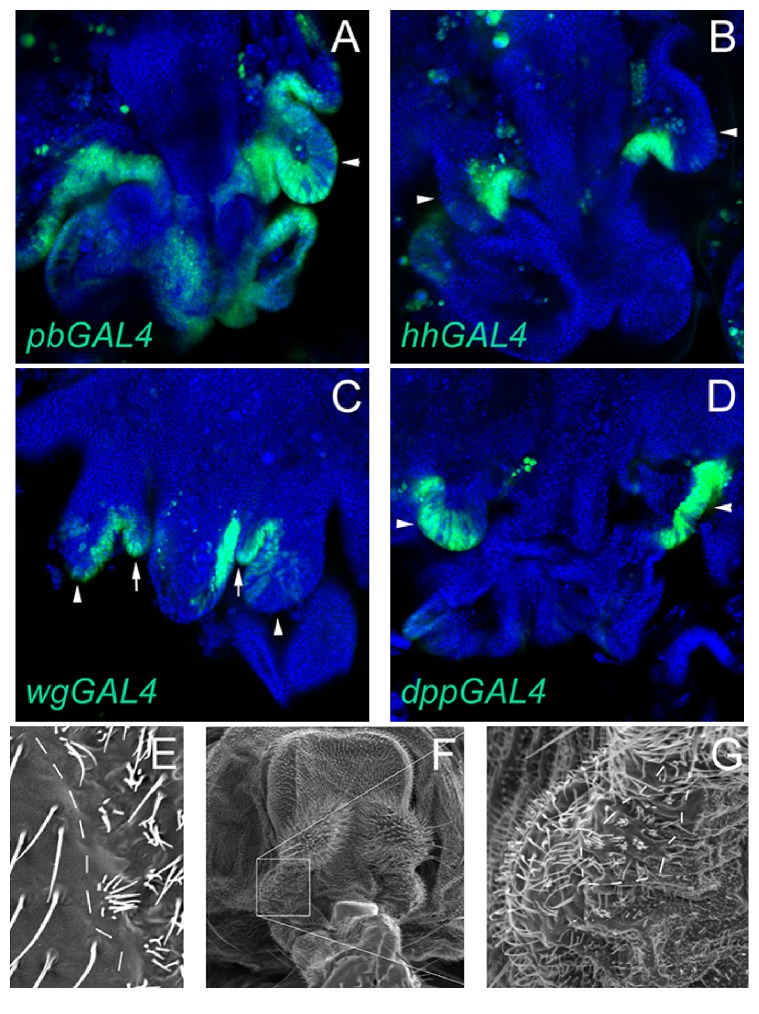
Expression of* pb*,* hh*,* wg*, and* dpp*-*GAL4* drivers during maxillary palpus development. In panels (A)–(D), the driver is indicated on the bottom lefthand corner. The arrowheads indicate the developing maxillary palpus and the arrows in panel (C) indicate the developing lancinia. The tissue is stained with DAPI (blue), and the expression of the drivers was detected with a* UASEGFP* reporter (green). Panel (E) is the expression of* hh-GAL4* in the wing marked by expression of TRC^S292A  T453A^ from the* UAStrc*^*S292A T453A*^ reporter. The dotted line indicates the anterior-posterior compartment boundary, and multiple short bristles are observed in the posterior compartment. Panels (F) and (G) are the expression of* hh-GAL4* in the maxillary palpus marked by expression of TRC^S292A  T453A^ from the* UAStrc*^*S292A T453A*^ reporter. The box in (F) indicates the close-up shown in (G). The dotted line in (G) indicates the field of cells that have multiple short tricombs indicating expression of TRC^S292A  T453A^.

The three secreted proteins WG, DPP, and HH are required for establishing the proximal-distal axis of the leg, wing, and antenna. These three proteins are potential candidates for a PB-regulated factor secreted from cells within or close to the maxillary socket that promotes growth of the distal maxillary palp along the proximal-distal axis. The expression patterns of these secreted factors were assessed using GAL4 driver lines during metamorphosis (Figure 4) [22, 23, 31]. Both* hh-GAL4* and* wg-GAL4* were expressed strongly in cells outside the developing distal maxillary palp and less so in some of the distal maxillary palp cells (Figures 4(B) and 4(C)). However,* dpp-GAL4* was strongly expressed in the distal maxillary palp cells ruling out DPP as a candidate for the PB-regulated growth factor (Figure 4(D)).

Although the site of expression of* wg-GAL4* outside the distal maxillary palp is the developing lancinia, the site of* hh-GAL4* expression outside the distal maxillary palp is unclear. To mark the adult cells that had strongly expressed* hh-GAL4*, the* UAStricornered*^*S292A T453A*^ fusion gene was used [29]. The TRC^S292A  T453A^ protein inhibits TRC^+^ protein activity resulting in multiple short tricombs on each cell that express TRC^S292A  T453A^. This was most easily observed when expression of TRC^S292A  T453A^ was driven by* hh-GAL4* in the posterior compartment of the wing (Figure 4(E)). The cells of the anterior compartment had long single tricombs on each cell, but the cells of the posterior compartment expressing GAL4 had multiple short tricombs (Figure 4(E)). The tricombs of the distal maxillary palp were unaffected when TRC^S292A  T453A^ was expressed using the* hh-GAL4* driver. However, ventral cells of the maxillary socket had multiple short tricombs indicating high levels of TRC^S292A  T453A^ expression had occurred in these cells (Figures 4(F) and 4(G)).

### 3.5. Requirement of* wg* and* hh* for the Growth of the Distal Maxillary Palp

Both WG and HH are required for many processes at many stages of development. Particularly relevant to this study is the importance of WG expression for the establishment of the maxillary palp field during larval development [14]. Therefore, to target reduction of expression of WG and HH to the developing maxillary palpus, the* pb-GAL4* driver and* UASRNAi* lines were used. The use of the* pb-GAL4* driver restricted expression of RNAi molecules to the maxillary palpus during pupal development, and in* Drosophila* RNAi mediated reduction of expression is cell autonomous [21]. To increase the activity of GAL4 expressed from* pbGAL4*, the flies were grown at 29°C [32]. Three* HH RNAi* lines were obtained: two of which (ID# 1402 and 1403) were predicted to have one off target (CG4637); and one line (ID# 43255) was predicted to have five off targets (CG17450, CG32819, CG32820, CG8665, CG9934) [21, 33, 34]. The predicted off targets were not shared between the two constructs. As a result of the crossing scheme, females expressed Dicer from* UASdicer2*, but males did not. In Dicer expressing females, all* HH RNAi* lines exhibited a significant reduction in the length of the distal maxillary palp (Table 4). The only significant reduction observed in males, which do not express Dicer, was with RNAi line 1403, which showed the strongest effect in females. Two* WG RNAi* line were obtained (ID# 13351 and 39676). Both carried the same construct with no predicted off targets and showed a significant reduction in the length of the distal maxillary palp. Using RNAi lines to target the reduction of expression of components of the WG and HH signal transduction pathways, Armidillo (ARM) and Pangolin (PAN) of the WG pathway and Smoothened (SMO) of the HH pathway were shown to be required for distal maxillary palp growth (Table 4).

### 3.6. The Requirement of HH in the Growth of the Distal Maxillary Palp

**Figure 5:**
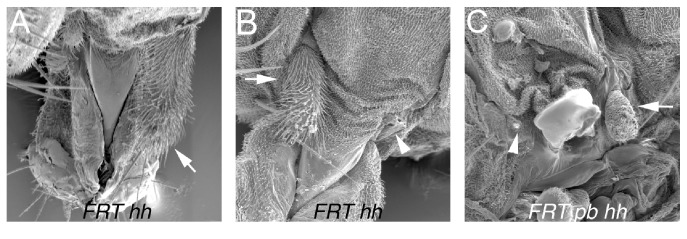
Genetic analysis of the requirement of* hh*. Panels (A) and (B) are scanning electron micrographs of the effects of clonal loss of HH function generated in flies with the genotype* y w; P*{*hspFLP*}*/*+*; P*{*ry*^*+*^*, neo*^*r*^*, FRT*}*82B e hh*^*9*^/*P*{*ry*^*+*^*, neo*^*r*^*, FRT*}*82B Sb*^*63b*^* M(3)95A*^*2*^* P*{*y*^*+*^*, ry*^*+*^}*96E*. The arrow in panel (A) points to a* hh* clone in the distal maxillary palp that was marked with Sb^+^ M^+^ bristles and that did not affect growth; the growth of the other three distal maxillary palps in panels (A) and (B) were affected to varying degrees and lacked bristles. Panel (C) is a* hh*^*9*^ genetic mosaic in a* pb* mutant background generated in the genotype* y w; P*{*hspFLP*}*/*+*; P*{*ry*^*+*^*, neo*^*r*^*, FRT*}*82B pb*^*27*^* Scr*^*2*^* e hh*^*9*^*/P*{*ry*^*+*^*, neo*^*r*^*, FRT*}*82B pb*^*20*^* Sb*^*63b*^* M(3)95A*^*2*^* P*{*y*^*+*^*, ry*^*+*^}*96E*. The arrows indicate reduced maxillary palpus and the arrowheads indicate the loss of the maxillary palpus in panels (B) and (C). In panel (C) the left vestigial maxillary palpus was missing and remaining vestigial palpus on the right was Sb M (*hh*^*9*^/+).

The* hh *gene encodes a secreted ligand, and therefore,* hh* mutant alleles behave noncell autonomously in a mosaic analysis [35]. To determine whether HH is required noncell autonomously for maxillary palpus development as expected from* hh-GAL4* expression (Figure 4), we induced* hh*^*9*^ mutant clones using FLP-mediated mitotic recombination. The right palp in Figure 5(A) had* hh*^*9*^ mutant clone of cells in the distal maxillary palp marked by the Sb^+^ bristles and exhibited a wild type phenotype indicating that* hh* was not required in the cells of the distal maxillary palp for growth. All other distal maxillary palps shown in Figures 5(A) and 5(B) were shortened or absent confirming the RNAi results that HH was required for distal maxillary palp growth. These two observations also show that HH was required noncell autonomously for distal maxillary palp growth.

Although both* pb* and* hh* were required for the growth of the distal maxillary palp, the pb and hh phenotypes were distinct: loss of PB expression resulted in a vestigial maxillary palpus; whereas, loss of HH expression resulted sometimes in a complete deletion of the maxillary palpus (Figures 5(A) and 5(B)). Using* FRT82 pb*^*27*^* Scr*^*2*^* hh*^*9*^ and* FRT82 pb*^*20*^* Sb M y*^*+*^ chromosomes,* hh*^*9*^ clones were induced in a* pb* mutant background (Figure 5(C)). As observed with* hh*^*9*^ clones in a wild type background,* hh*^*9*^ clones in a* pb* mutant background also resulted in loss of the vestigial palp indicating that growth of the vestigial palp is HH-dependent (Figure 5(C)).

### 3.7. Expressions of* wg-GAL4* and* hh-GAL4* Were Not PB-Dependent

**Figure 6:**
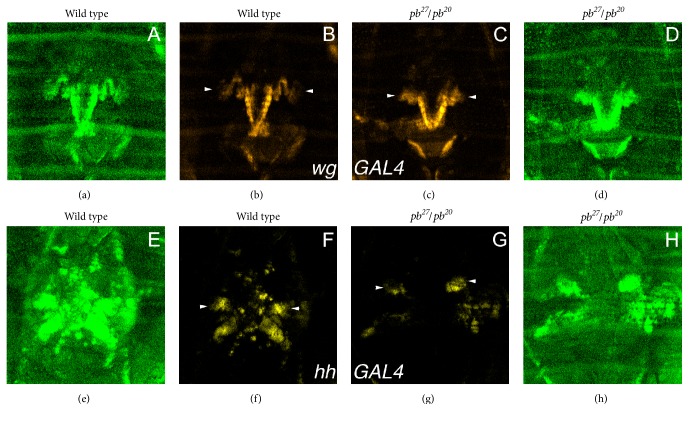
Expression of* wg-GAL4* and* hh-GAL4* in wild type and* pb*^*27*^*/pb*^*20*^ mutants. Panels (A)–(D) are* wg-GAL4*; panels (E)–(H) are* hh-GAL4*. Panels (A), (D), (E), and (H) show expression of* UbiGFP* (green). Panels (B), (C), (F), and (G) show expression of YFP (yellow) from a* UASYFP* reporter gene. Panels (A), (B), (E), and (F) are wild type and panels (C), (D), (G), and (H) are* pb*^*27*^*/pb*^*20*^ mutants. The arrowheads indicate expression of YFP in the maxillary palps of wild type and* pb* mutants at 16 h AHE.

**Figure 7:**
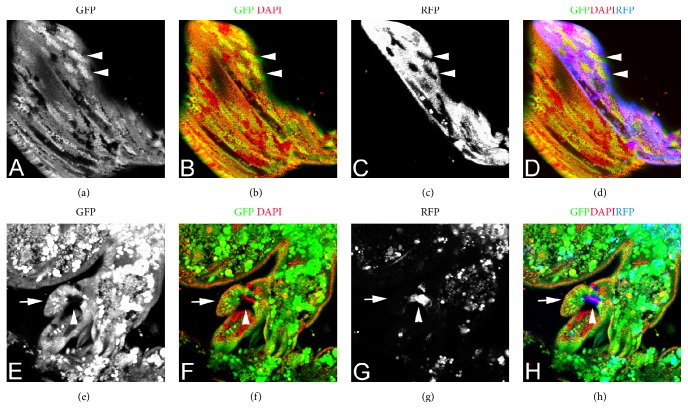
Clonal analysis of the requirement of PB in* hh-GAL4* expression. All clones were generated in the genotype* y w; P*{*UASRFP*}*/P*{*hspFLP*}*; P*{*UAStrc*^*S292A T453A*^}*, FRT82B pb*^*27*^*hh-GAL4/FRT82B P*{*UbiGFP*}. Panels (A)–(D) are a pupal wing and panels (E)–(H) are a pupal maxillary palp. Panels (A) and (E) are GFP expression; panels (B) and (F) are GFP expression (green) and nuclei visualized with DAPI (red); panels (C) and (G) are RFP expression; and panels (D) and (H) are GFP (green) and RFP (blue) expression with the nuclei visualized with DAPI (red). The two arrowheads in panels (A)–(D) indicate clones of cells that are homozygous for* UbiGFP* and have lost RFP expression due to loss of* hh-GAL4*. In panels (E)–(H), the arrow indicates the developing distal maxillary palp, and the arrowhead indicates a* pb*^*27*^ mutant clone that shows strong expression of RFP indicating strong expression of* hh-GAL4*.

The expression of* wg-GAL4* and* hh-GAL4* were assessed in parallel live imaging experiments where both wild type and* pb*^*27*^*/pb*^*20*^ prepupae were mounted side by side and allowed to undergo metamorphosis. The expression of GAL4 was detected with a* UASYFP* reporter gene and all cells of the pupae were marked with GFP expressed from* UbiGFP*. In parallel live imaging of* wg-GAL4* expression in wild type and* pb*^*27*^*/pb*^*20*^ prepupae and pupae,* wg-GAL4* was strongly expressed in lancinia of both the wild type and* pb* mutant (Figure 6; Supplemental Data Movie 3) indicating that* wg* is not regulated by PB. In parallel live imaging of* hh-GAL4* expression in wild type and* pb*^*27*^*/pb*^*20*^ prepupae,* hh-GAL4* was strongly expressed in the salivary glands of wild type but not* pb*^*27*^*/pb*^*20*^ prepupae indicating that* hh* expression in the salivary gland is PB-dependent (Supplemental Data Movie 4). However, in wild type pupae* hh-GAL4* was expressed strongly in the cells of the maxillary socket and* hh-GAL4* expression was only expressed a little less in the maxillary palp socket cells of the vestigial maxillary palpus of* pb* mutants (Figure 6 Supplemental Data Movie 4). Although in other repeat experiments, a greater difference between expression of* hh-GAL4* in wild type and* pb* mutants was observed,* hh-GAL4* is still expressed in* pb* mutants. The variation observed between experiments could be due to the* pb* mutant mouthpart cells not being very healthy resulting in a nonreproducible level of* hh-GAL4* expression. Or as clearly observed in the movies, once the maxillary palps of wild type and* pb* mutants start to differentiate they are very different from one another early in differentiation and the lower level of* hh-GAL4* expression may reflect divergence of the structure of the wild type and mutant palps. To investigate further whether PB was required for* hh-GAL4* expression, a FLP-mediated mosaic analysis was performed. The* hh-GAL4* allele is an insertion of a GAL4 enhancer detector into the* hh* locus, and both the* pb* and* hh* loci are on the right arm of chromosome 3. Therefore, the* FRT82 pb*^*27*^* hh-GAL4* chromosome created to perform the mosaic analysis resulted in 3 distinct cellular genotypes that were most easily observed in the posterior compartment of the wing (Figures 7(A)–7(D)): parental RFP and GFP expressing cells, loss of GFP expression (*pb*^*27*^*hh-GAL4*) but not RFP expression, and gain of GFP expression but loss of RFP expression due to the loss of* hh-GAL4 (FRT UbiGFP)*. In* pb*^*27*^ clones in the maxillary socket cells, RFP, and therefore* hh-GAL4*, was still expressed at a high level (Figures 7(E)–7(H)). PB is not required cell autonomously for* hh-GAL4* expression.

## 4. Discussion

### 4.1. Noncell Autonomous Requirement of PB in Maxillary Palpus Development

PB is required for the development of both the adult proboscis and the maxillary palpus [3]. At the wandering third instar larval stage, PB is expressed in the labial imaginal disc but not in the eye antennal imaginal disc that harbors the primordia for the maxillary palpus [20]. During the prepupal stage* pb-GAL4* expression intensifies in the differentiating labial discs, and is expressed in the maxillary palpus primordia. During the first thirty hours of pupal development the mouthparts undergo major events of morphogenesis forming a structure that is easily recognized as adult mouthparts. During this stage of pupal development* pb-GAL4* expression is dynamic and intense. The expression of PB and* pb-GAL4* are not restricted to the proboscis and distal maxillary palp, but are also expressed in the cells of the maxillary socket and surrounding tissue. Expression of PB in the cells of the maxillary socket, or cells close to it, is required for the growth of the distal maxillary palp. This noncell autonomous requirement of PB in cells outside the distal maxillary palp is not due to the transcriptional regulation of genes that encode the growth factors HH and WG, even though WG and HH are required for growth of the distal maxillary palp.

Although the HOX protein PB is a transcription factor, and is expected to have a cell autonomous role in regulation of PB-regulated genes, these regulated genes can function on pathways involved in cell-cell communication. This phenomenon is well described in a number of HOX systems in* Drosophila*. In morphogenesis of the embryonic gut, Ultrabithorax (UBX) is required for the expression of the growth factor DPP [36]. Also SCR and other HOX proteins are required noncell autonomously for induction of ectopic tarsi, and Antennapedia is required noncell autonomously for leg determination [12, 37, 38]. PB is required in a complex combination of cell autonomy and noncell autonomy in the regulation of WG and HH pathways during proboscis determination [39, 40]. For regulation of the growth of the haltere UBX is required noncell autonomously and UBX regulated genes involved in mediating this noncell autonomy are identified [41, 42].

PB is also required in the distal maxillary palps cells for growth. In our mosaic analysis we were unable to assess the growth phenotype of* pb*^*+*^ and* pb*^−^ cells in palps of mixed* pb*^*+*^*/pb*^−^ genotypes, and therefore, we were unable to assess whether PB is required cell autonomously in the developing distal maxillary palp cells. If PB is required cell autonomously in the distal maxillary palp cells, then it is possible that PB also regulates the expression of the components (receptor, signal transduction, etc.) that receive the noncell autonomous PB-regulated signal coming from cells outside the distal maxillary palp.

### 4.2. PB and Proximal-Distal Axis Formation

The major maxillary palpus phenotype caused by loss of PB expression is the loss of growth of the distal maxillary palp along the proximal-distal axis resulting in a vestigial stump. This phenotype suggested the possibility that PB regulates the expression of genes required for the formation of the proximal-distal axis of the leg, wing and antennal appendages.* wg* and* hh* are transcribed in cells outside the distal maxillary palp, and are required for the growth of the distal maxillary palp, but the transcription of these genes does not require PB. These results may suggest that the system PB regulates for proximal-distal axis formation is independent of the system that WG and HH function in for proximal-distal axis formation of the distal maxillary palp. Although this may be the case, our results really only suggest that transcription of the genes that encode the secreted ligands WG and HH are not PB-regulated. UBX is required to suppress the growth of haltere cells, and UBX does not do this by suppressing DPP expression directly but through components involved in the interpretation of the DPP gradient [41, 42]. It is possible that PB functions in a similar manner during distal maxillary palp growth. For example, PB may regulate the expression of a gene in cells outside the distal maxillary palp that is required for a specific posttranslational modification of the secreted factor WG or HH, and this modified form of WG or HH is important for proximal-distal axis formation [43]. In a second explanation, PB is required for repression of expression of a secreted inhibitor of a growth factor. Therefore, PB may have a role in regulating the activity of HH, WG, or DPP in proximal-distal axis formation.

### 4.3. The Derived* Drosophila* Maxillary Palpus

The maxillary palpus of* Drosophila* is a highly derived structure relative to that proposed for the archetypical insect head [1, 44, 45]. This high level of derivation may be reflected in two other observations. First, analysis of mitotic clones did not detect anterior-posterior compartment formation in the maxillary palpus even though HH is expressed in a spatially restricted domain during maxillary palpus differentiation [30, 46]. Second,* Dll-GAL4* expression in the distal maxillary palp and maxillary socket [17] suggests that the derived maxillary palpus may be of telopodite origin. These two observations may suggest that the compartmental boundaries are established during metamorphosis and the maxillary palpus is homologous to the distal arista and tarsus of the antenna and leg, respectively. Therefore, PB may be regulating the Epidermal Growth Factor Receptor (EGFR) pathway, which is important for determining the proximal-distal axis of the distal segments of the legs and antennae [47, 48]. This is supported by the identification of components of the EGFR signal transduction pathway as being important for the antenna to maxillary palp transformation caused by ectopic expression of PB [49]. In addition, analysis of the pathways involved in proximal-distal axis formation of* Tribolium castaneum* mouthparts has shown an involvement of the EGFR pathway [45]. Although an interesting possibility, when considering the ligands of the conserved genetic toolkit, there is unlikely to be a single cell that is unaffected by the HH, WG, EGFR, Notch etc. pathways during their development, so it may be naïve to look for direct PB-dependent regulation of the genes that encode the secreted ligand of these pathways as HOX proteins may regulate growth by more subtle mechanisms [41, 42].

## 5. Conclusions

The HOX transcription factor PB is required both in the cells of the distal maxillary palp and in cells of, or close to, the adjacent maxillary socket for growth of the distal maxillary palp. Therefore, an important role of PB in the growth of the distal maxillary palp is the regulation a cell-cell communication pathway(s). The genes* wg* and* hh* are expressed in cells outside the distal maxillary palp and are required for growth of the maxillary palp. Although WG and HH are good candidates for mediating the noncell autonomous requirement of PB, transcription of the* wg* and* hh* genes is not directly regulated by PB. But the option remains that PB may be required for activation of either WG or HH protein activity, or that PB may regulate the expression of another signaling pathway altogether.

## Supplementary Material

Movie 1. Live imaging of pb-GAL4 expression during metamorphosis. The anterior end of prepupa/pupa is at the top. The time stamp gives time in hours: minutes: seconds BHE or AHE.
Movie 2. Dual live imaging detecting pb-GAL4 expression using a UASYFP reporter gene (right) and UbiGFP expression (left). The anterior of the pupa is at the top of the frames. The time stamp gives time in hours: minutes: seconds BHE or AHE. The <mx indicate the maxillary palpus AHE.
Movie 3. Dual live imaging detecting wg-GAL4 expression using a UASYFP reporter gene (center images) and UbiGFP expression (flanking images) in wild-type (left) and pb27/pb20 mutants (left). The anterior of the pupae are at the top of the frames. The time stamp gives time in hours: minutes: seconds BHE or AHE.
Movie 4. Dual live imaging detecting hh-GAL4 expression using a UASYFP reporter gene (center images) and UbiGFP expression (flanking images) in wild-type (left) and pb27/pb20 mutants (left). The anterior of the pupae are at the top of the frames. The time stamp gives time in hours: minutes: seconds BHE or AHE.

## Figures and Tables

**Table 1:** Stocks.

Name	Genotype	Origin
APS303	*y w; P*{*hspFLP*}*; pb*^*20*^*/TM6B, P*{*w*alL*y*}	[12]
APS304	*y w, P*{*w*^*+*^*, pb*^*a*^*>y*^*+*^*>Tubα1*}*B; pb*^*27*^*/TM6B, P*{*w*alL*y*}	[12]
APS202	*y w; P*{*ry*^*+*^*, neo*^*r*^*, FRT*}*82B pb*^*27*^*/TM6B, P*{*w*alL*y*}	[12]
APS205	*y w; P*{*ry*^*+*^*, neo*^*r*^*, FRT*}*82B Sb*^*63b*^* M(3)95A*^*2*^* P*{*y*^*+*^*, ry*^*+*^}*96E/TM6B, P*{*w*alL*y*}	This work
APS201	*y w; P*{*ry*^*+*^*, neo*^*r*^*, FRT*}*82B pb*^*27*^* Scr*^*2*^* P*{*w*^*+*^*, ry*^*+*^}*90E/TM6B, P*{*w*alL*y*}	[12]
APS121	*y w; P*{*ry*^*+*^*, neo*^*r*^*, FRT*}*82B pb*^*20*^* Sb*^*63b*^* M(3)95A*^*2*^* P*{*y*^*+*^*, ry*^*+*^}*96E/TM6B, P*{*w*alL*y*}	[12]
DJ103	*y w; P*{*ry*^*+*^*, neo*^*r*^*, FRT*}*82B M(3)95A*^*2*^* P*{*y*^*+*^*, ry*^*+*^}*96E P*{*exd*^*+*^*, w*^*+*^}*/TM6B, P*{*w*alL*y*}	[19]
GS902	*y w, P*{*hspFLP*}^*122*^*; P*{*UAStrc*^*S292A T453A*^*,w*^*+*^}*, hh-GAL4/TM2*	G. Struhl
APS402	*y w; P*{*UAStrc*^*S292A T453A*^*,w*^*+*^}*, P*{*ry*^*+*^*, neo*^*r*^*, FRT*}*82B pb*^*27*^* hh-GAL4/TM6B, P*{*w*alL*y*}	This work
APS403	*y w; P*{*UASmyr-mRFP, w*^*+*^}*/CyO; P*{*UAStrc*^*S292A T453A*^*,w*^*+*^}*, P*{*ry*^*+*^*, neo*^*r*^*, FRT*}*82B pb*^*27*^* hh-GAL4/TM6B, P*{*w*alL*y*}	This work
APS404	*y w; P*{*UASEGFP, w*^*+*^}*, pb*^*20*^*/TM6B, P*{*w*alL*y*}	This work
APS405	*y w; P*{*ry*^*+*^*, neo*^*r*^*, FRT*}*82B pb*^*27*^* Scr*^*2*^* e hh*^*9*^*/TM6B, P*{*w*alL*y*}	This work
CB10	*w1118; P*{*pb-GAL4, w*^*+*^}*, P*{*UASlacZ, w*^*+*^}*/CyO*	[20]
GS30	*w* ^*1118*^ *; P*{*ry*^*+*^*, neo*^*r*^*, FRT*}*82B e hh*^*9*^*/TM2*	G. Struhl
GFP	*w* ^*1118*^ *; P*{*UASEGFP, w+*}	Bloomington stock center
VDRC60010	*w* ^*1118*^ *, P*{*UASdicer2, w*^*+*^}*; Pin/CyO*	[21]
GP1	*y w; P*{*UASYFP, w*^*+*^}*; P*{*Ubi GFP*}	This work
APS454	*y w; P*{*UAS YFP, w*^*+*^}*; P*{*ry*^*+*^*, neo*^*r*^*, FRT*}*82B pb*^*27*^*/TM6B, P*{*w*alL*y*}	This work
APS455	*y w; pb* ^*20*^ *, P*{*Ubi GFP, w*^*+*^}	This work
KB1	*y w, P*{*hspFLP*}*; wgGAL4*^*270*^	[22]
S491	*w* ^*1118*^ *;P*{*dpp-GAL4, w+*}	[23]

**Table 2:** Distribution of phenotypes in the *pb* loss-of-function mosaic analysis.

Sb M y^+^*(pb*^*+*^)		Sb^+^ M^+^ y^−^*(pb*^−^)	
Wild type maxillary palps	Reduced maxillary palps	Wild type maxillary palps	Reduced maxillary palps
40^a^	13	0	96

^a^The numbers only include distal maxillary palps that were completely Sb^+^ or Sb; distal maxillary palps that were a mix of genotypes were not included.

**Table 3:** Distribution of phenotypes in PB ectopic expression rescue mosaic analysis.

y^−^*(pb*^*+*^)		y^+^*(pb*^−^)	
Rescued maxillary palps	Reduced maxillary palps	Rescued maxillary palps	Reduced maxillary palps
63^a^	10	0	51

^a^The numbers only include distal maxillary palps that were completely y^+^ or y^−^; distal maxillary palps that were a mix of genotypes were not included.

**Table 4:** The effect of mouthpart-specific, RNAi mediated inhibition of components of the WG and HH pathways on distal maxillary palp length.

RNAi line	Construct	Targeted mRNA	Length of the distal maxillary palp (*μ*m) ± SEM^*∗*^	
			Female *(UASdicer2*^*+*^)	Male *(UASdicer2*^−^)
*y w*	—	—	160 ± 3^a^ (5)^#^	139 ± 1^a^ (5)
1402	193	*HH*	144 ± 3^b^ (5)	141 ± 2^a^ (5)
1403	193	*HH*	126 ± 1^c^ (5)	127 ± 3^b^ (5)
43255	6242	*HH*	138 ± 2^b^ (5)	136 ± 3^a^ (5)

*y w*	—	—	156 ± 3^a^ (5)	134 ± 1^a^ (5)
13351	5007	*WG*	134 ± 3^b^ (5)	135 ± 4^a^ (5)
39676	5007	*WG*	130 ± 6^b^ (2)	127 ± 2^a^ (3)
7767	1372	*ARM*	138 ± 1^b^ (5)	141 ± 1^a^ (4)
107344	102545	*ARM*	— (0)	108 ± 8^b^ (4)
25940	10429	*PAN*	143 ± 3^b^ (5)	138 + 2^a^ (5)
9542	577	*SMO*	136 ± 2^b^ (4)	134 ± 2^a^ (5)

^*∗*^Data in the same column with the same letter are not significantly different (*P *> 0.05).

^#^Number of biological replicates.
